# Short term safety of *bulbine natalensis* supplementation in healthy men

**DOI:** 10.1186/1550-2783-9-S1-P33

**Published:** 2012-11-19

**Authors:** Jennifer E Hofheins, Scott M Habowski, Tim N Ziegenfuss, Hector L Lopez

**Affiliations:** 1 The Center for Applied Health Sciences, Stow, OH, USA

## Background

*Bulbine natalensis* is a perennial herb indigenous to South Africa that is currently being marketed as a prosexual product for men. As the first step in a series of experiments designed to assess in the *in vivo* effects of *Bulbine natalensis* (ProLensis™) supplementation, we performed a placebo-controlled, double-blind clinical trial to assess the short-term safety of this ingredient.

## Methods

After giving informed consent and being cleared for participation by passing a screening physical and EKG, 36 apparently healthy men (mean ± SD age, height, weight: 29.4 ± 7.7 y, 177.2 ± 5.2 cm, 82.2 ± 10.7 kg) consumed 4 capsules of ProLensis™ (325 mg in the morning, 325 mg six hours later) or a matched placebo every day for 28 days. Clinical chemistry panels (renal, hepatic, and hematological biomarkers) and general markers of health (heart rate, blood pressure, EKG) were assessed before and after 28 days of supplementation. Data were analyzed via ANCOVA using baseline values as the covariate and statistical significance was set *a priori* at P≤0.05.

## Results

In 27 of 29 variables, no differences were noted between groups. Alkaline phosphatase (AP) increased marginally in the ProLensis™group (+2.0 IU/L, +3%) compared to a parallel decrease the Placebo group (-2.4 IU/L, -3.8%); P<0.04. In contrast, creatinine (Creat) decreased slightly in the ProLensis™group (-0.08, -7.4%) compared to no change in the Placebo group (P<0.003). It is our opinion that the observed differences in AP and Creat are not clinically relevant given that all values for both groups fell well within normative clinical limits (i.e. typical values for AP range from 20 to 140 IU/L^1^; typical values for Creat range from 0.6 to 1.3 mg/dL for men and 0.5 to 1.1 mg/dL for women^2^).

## Conclusions

Within the confines of the current experimental design (i.e. subject demographics, dose and duration of use) these preliminary data suggest that ProLensis™is as safe as Placebo with respect to the hemodynamic, hepatic, renal, and hematologic biomarkers assessed. Future studies should seek to clarify extraction methods and bioactive(s), investigate potential efficacy, and confirm these safety data to strengthen the total body of evidence.

